# The plant nuclear lamina proteins NMCP1 and NMCP2 form a filamentous network with lateral filament associations

**DOI:** 10.1093/jxb/erab243

**Published:** 2021-06-04

**Authors:** Kiyoshi Masuda, Riku Hikida, Kaien Fujino

**Affiliations:** 1 Laboratory of Crop Physiology, Research Faculty of Agriculture, Hokkaido University, Kita 9 Nishi 9, Sapporo 060-8589, Hokkaido, Japan; 2 Ohio State University, USA

**Keywords:** Coiled-coil protein, filamentous network, NMCP1, NMCP2, nuclear lamina, self-assembly, stimulated emission depletion microscopy, super-resolution microscopy

## Abstract

Plant genomes lack genes encoding intermediate filament proteins, including lamins; however, functional lamin analogues are presumed to exist in plants. Plant-specific coiled-coil proteins, that is, nuclear matrix constituent proteins (NMCPs), are the most likely candidates as the structural elements of the nuclear lamina because they exhibit a lamin-like domain arrangement. They are exclusively localized at the nuclear periphery and have functions that are analogous to those of lamins. However, their assembly into filamentous polymers has not yet been confirmed. In this study, we examined the higher-order structure of NMCP1 and NMCP2 in *Apium graveolens* cells by using stimulated emission depletion microscopy combined with immunofluorescence cell labelling. Our analyses revealed that NMCP1 and NMCP2 form intricate filamentous networks, which include thick segments consisting of filament bundles, forming a dense filamentous layer extending across the nuclear periphery. Furthermore, the outermost chromatin distribution was found to be in the nucleoplasm-facing region of the nuclear lamina. Recombinant *Daucus carota* NMCP1 with a His-tag produced in *Escherichia coli* refolded into dimers and self-assembled into filaments and filament bundles. These results suggest that NMCP1 and NMCP2 organize into the nuclear lamina by forming a filamentous network with filament bundles that localize at the nuclear periphery.

## Teaser

Super-resolution microscopy revealed that the plant nuclear lamina proteins NMCP1 and NMCP2 organize into filamentous networks with filament bundles.

## Introduction

In eukaryotic cells, the nuclear envelope (NE) is a highly specialized membrane that compartmentalizes the nucleoplasm from the cytoplasm. The NE of metazoans is composed of three fundamental domains: two nuclear membranes called the inner and outer nuclear membranes, nuclear pore complexes, and a nuclear lamina (NL) (Hetzer, 2010). The NL is a filamentous layer lying at the nucleoplasmic face of the inner nuclear membrane and consists of type V intermediate filament (IF) proteins termed lamins (Aebi *et al.*, 1986; McKeon *et al.*, 1986; Steinert and Roop, 1988). Lamins provide mechanical stability, organize chromatin, and regulate transcription, replication, and nuclear positioning (Dechat *et al.*, 2010; Burke and Stewart, 2013). In humans, mutations in the gene encoding lamin A/C cause a broad range of systemic diseases such as laminopathies and cancers (Mattout *et al.*, 2006; Zuela *et al.*, 2012). Comparative analyses of lamins across eukaryotes have revealed that lamin genes are found in highly divergent taxa, including lower eukaryotes (Kollmar, 2015; Koreny and Field, 2016).

NL-like structures have been identified in several plant species by using electron microscopy (Galcheva-Gargova *et al.*, 1988; Moreno Díaz de la Espina *et al.*, 1991; Masuda *et al.*, 1993), following a methodology similar to that used to visualize the nuclear matrix of rat liver cell nuclei (Dwyer and Blobel, 1976). However, the existence of lamins or lamin-related IF proteins in plants has long been debated (Blumenthal *et al.*, 2004; Fiserova *et al.*, 2009; Koreny and Field, 2016) because no genes encoding canonical IF proteins, including lamins, have been detected in the *Arabidopsis thaliana* genome. Furthermore, genes encoding lamins and IF proteins have not been confirmed in any of the genome assemblies of plants or rhodophyte expressed sequence tag/cDNA libraries (Kollmar, 2015). In contrast, a candidate protein having an NL-like structure, nuclear matrix constituent protein 1 (NMCP1), was identified in carrot cells by using immunological approaches (Masuda *et al.*, 1993, 1997).

In angiosperms, the *NMCP* genes can be classified into two families: type 1 *NMCP*s (including *NMCP1* and its paralogs) and type 2 *NMCP*s (Ciska and Moreno Diaz de la Espina, 2013; Wang *et al.*, 2013; Ciska *et al.*, 2018). Although terrestrial algae have genes phylogenetically distant from those of embryophyte *NMCP*s, green algae (chlorophytes) do not have the corresponding genes (Wang *et al.*, 2013; Ciska *et al.*, 2019). Phylogenetic analyses have suggested that *NMCP* progenitor genes emerged in terrestrial (Charophyta) algae and diverged into the *NMCP1* and *NMCP2* gene families at the time of the emergence of seed plants (Ciska *et al.*, 2019). A previous study showed that carrot NMCP1 (DcNMCP1) exhibits moderate sequence similarity to myosin, tropomyosin, and some IF proteins (Masuda *et al.*, 1997).

Arabidopsis orthologs of carrot NMCPs were identified by analysing hypothetical proteins exhibiting lamin-like tripartite structures with central coiled-coil domains (Rose *et al.*, 2004; Meier, 2007). The *NMCP1*-related Arabidopsis genes, *CRWN1* to *CRWN4* (originally named *LINC1* to *LINC4*), were identified and characterized by phenotypic analyses of loss-of-function mutants (Dittmer *et al.*, 2007; Wang *et al.*, 2013). The phylogenetic tree indicates that *CRWN*s are divided into two gene subfamilies: the *CRWN1* clade includes two paralogous genes, *CRWN2* and *CRWN3*, whereas *CRWN4* belongs to the *NMCP2*-type clade (Wang *et al.*, 2013; Poulet *et al.*, 2016; Gumber *et al.*, 2019). CRWN1 and CRWN4 are localized at the nuclear periphery, whereas CRWN2 and CRWN3 reside in the nuclear interior (Dittmer *et al.*, 2007; Sakamoto and Takagi, 2013); the localization of CRWN2 and CRWN3 at the nuclear periphery has been reported recently (Sakamoto *et al.*, 2020). A recent study showed that CRWN1 is more similar to paramyosin/myosin than to lamins (Gardiner *et al.*, 2011). However, the mechanism by which the coiled coils of the NMCP precursor establish a tripartite structure, including the amino-terminal domain and carboxy-terminal domain (CTD), is not yet known.

At present, NMCP1/CRWN1 is considered to be a lamina protein and a functional lamin analogue in plants, suggesting that CRWN1 functions in the regulation of nuclear morphology and positioning (Tamura *et al.*, 2013) and in plant development (Dittmer *et al.*, 2007; Wang *et al.*, 2013). Recent studies have suggested that it also functions in chromatin anchoring and organization at the nuclear periphery, protection against biotic/abiotic stresses, and the systemic acquired resistance signal transduction pathway (Meier, 2016; Choi *et al.*, 2019; Hu *et al.*, 2019; Wang *et al.*, 2019; Sakamoto *et al.*, 2020).

The high probability of NMCPs forming coiled coils and lamin-like domain arrangement strongly suggests their higher-order assembly into filamentous polymers. However, this prediction has not been experimentally confirmed. Therefore, elucidating the structural organization of NMCP proteins is a crucial step in validating the NL structure and elucidating its functions in plants. In this study, we analysed the higher-order structure of NMCP1 and NMCP2 by using stimulated emission depletion (STED) microscopy, which is a type of super-resolution microscopy (SRM) (Wegel *et al.*, 2016; Schermelleh *et al.*, 2019). Recent theoretical and technical innovations based on different principles have led to the development of light microscopes that can break the diffraction limit (Huang *et al.*, 2010; Schermelleh *et al.*, 2010). This study aimed to elucidate whether NMCP1 and NMCP2 polymerize to form a filamentous assembly and how they are organized into the NL.

## Materials and methods

### Plant sources and cell cultures

The cell line AG3, established from *Apium graveolens* (Umbelliferae) hypocotyl tissues, was used in the experiments. The cells were cultured in a modified Murashige and Skoog medium (Murashige and Skoog, 1962) supplemented with 50 μM 2,4-dichlorophenoxyacetic acid. The culture flasks were placed on a shaking platform (Kühner AG, Birsfelden, Switzerland) with a reciprocal motion of 50 mm at 82 rpm. The cells were maintained by transferring a 4 ml aliquot to 91 ml of fresh medium at 14-day intervals.

### Antibodies

We used mAbCML-1 (anti-DcNMCP1) and mAbCML-10 (anti-DcNMCP2) monoclonal antibodies developed in-house (Masuda *et al.*, 1993); they react specifically with a wide range of NMCP1 and NMCP2 proteins, respectively, from Umbelliferae species. For immunofluorescence labelling, Alexa Fluor-555-conjugated goat anti-mouse IgG (H+L), Alexa Fluor-488-conjugated goat anti-mouse IgG (H+L) (both from Molecular Probes-Thermo Fisher Scientific, Eugene, OR, USA), and alkaline phosphatase-conjugated goat anti-mouse IgG (H+L) (Sigma-Aldrich, St. Louis, MO, USA) were used as secondary antibodies.

### Cell preparation and immunofluorescence labelling

Cells were harvested during the exponential growth phase (4–7 days after transfer). Cell dissociation was facilitated by partially digesting the cell walls with a solution containing 2% (w/v) Cellulase ‘Onozuka’ R-10 and 0.02% (w/v) Pectolyase Y-23 (Wako Pure Chemicals, Osaka, Japan) in 0.35 M sorbitol, 2% (w/v) bovine serum albumin (BSA) fraction V (Roche, Basel, Switzerland), and a protease inhibitor mixture (Nacalai Tesque, Kyoto, Japan) for 30 min at 25 °C with gentle agitation. The cells were washed once with 20 mM piperazine-1-4-bis-ethanesulfonic acid (MES)-KOH (pH 5.8) containing 0.35 M sorbitol, 2 mM CaCl_2_, 3 mM MgCl_2_, 50 mM KCl, and a protease inhibitor mixture (Nacalai Tesque). The cells were then immobilized on cover slips (0.16–0.19 mm thickness; No.1-S, Matsunami Glass, Kishiwada, Japan), fixed chemically at 0 °C for 40 min with 2.0% (v/v) paraformaldehyde in 20% (v/v) ethyl alcohol, and permeabilized with 0.2% (w/v) Triton X-100 in phosphate buffered saline. The fixed cells on the cover slips were then incubated in a blocking buffer containing 40 mM MES-KOH (pH 6.4), 100 mM KCl, and 2% (w/v) BSA for 2 h at 25 °C.

The cells were stained using a conventional indirect immunofluorescence method and/or the ZENON labelling method (Molecular Probes-Thermo Fisher Scientific). For ZENON labelling, mAbCML-1 or mAbCML-10 were previously conjugated with Alexa Fluor dye-conjugated Fab fragments (ZENON fragments) by using a labelling kit (Molecular Probes-Thermo Fisher Scientific) and used for cell labelling, according to the manufacturer’s instructions. For double labelling with mAbCML-1 and mAbCML-10, cells were stained with a combination of ZENON-based and indirect immunofluorescence staining techniques. After staining, the cells were rinsed several times with 40 mM Na phosphate buffer (pH 6.0) and embedded in the anti-fade mounting medium Prolong Diamond (Molecular Probes-Thermo Fisher Scientific) by placing the cover slips upside down on slides at room temperature (18–23 °C) overnight.

### DNA staining

DNA was stained with SYBR Green I (5760A; Takara Bio Inc., Kusatsu, Japan) at 1:10 000 dilution in 40 mM Na phosphate buffer (pH 6.0), because 4,6-diamidino-2-phenylindole and Hoechst dyes are incompatible with STED microscopy.

### STED microscopy

A Leica TCS SP8 STED 3× system driven by LASX software (Leica Microsystems, Wetzlar, Germany) was used for both STED microscopy and confocal laser scanning microscopy (CLSM). The microscope was operated at room temperature (21±2 °C).

A regular photomultiplier tube was used as the confocal detector. An avalanche photodiode was used as a highly sensitive detector for STED microscopy. Optics with apochromatic objectives, including a 100×/NA 1.40 oil immersion objective lens, were routinely used for image capturing.

The relative intensity of the second laser (STED laser) was adjusted to 30–50% of the excitation laser power. The intensity of the 3D STED (z-STED) laser was adjusted to 20–40% of the excitation laser power. Time-gated detection, which effectively rejects photons emitted by short-lived excited-state fluorophores and selectively captures those emitted by long-lived excited-state fluorophores, was opened between 0.5 ns and 6.5 ns after applying the excitation pulse.

### Image processing and measurements

Recorded STED image data were deconvoluted by a function of Huygens v.15.01, with a signal-to-noise ratio of 7 and <40 iterations (default, 40 iterations). Image processing, including patterning, skeletonizing, creating hue–saturation–brightness (HSB) thresholding; binary imaging; making line and surface plots; and size measurements were performed using ImageJ v.1.46 software (National Institutes of Health, Bethesda, MD, USA) running on a Mac OS 10.5.8 operating system (Apple Inc., Cupertino, CA, USA).

The filamentous networks were characterized by extracting skeletal elements from the immunofluorescence images. The skeletons were produced by applying histogram equalization and gamma correction (applied at a value between 1.8 and 2.2) to adjust the contrast; areas of intermediate brightness between contours determined using the Over/Under Threshold tool in the Adjust menu were selected and extracted, the greyscale image was converted to a binary, and the pattern was thinned to single-pixel lines.

### Expression of His-tagged DcNMCP1 and DcNMCP2 in *Escherichia coli*

The expression vectors encoding DcNMCP1 and DcNMCP2 with 6× His-tags (consisting of six histidine residues) were constructed using pET6×HN-C (Clontech Lab Inc., Mountain View, CA, USA) as the backbone plasmid. The plasmid was digested with *Nco*I and *Sal*I (New England Biolabs, Ipswich, MA, USA) to yield a cloning site for N-terminal 6× His-tagged DcNMCP1 expression. The longest ORF fragment of *DcNMCP1* cDNA was amplified from the cDNA clone JL2603 by using PCR with the primers 5′-ATGTTTACTCCACCAAAGAAG-3′ and 5′-TCATCAAGTTGTGAGGAACTTC-3′. Next, the sequence encoding a 6× His tag was added to the fragment by using PCR with the primers 5′-CATCACCATCACCATCACGGTATGTTTACTCCACCAAAGAAG-3′ and 5′-TCTGGTCGACTCATCAAGTTGTGAGGAACTTC-3′, resulting in a 5′-extension encoding 6× His and a 3′-extension with a *Sal*I restriction site after the intrinsic termination codon. The PCR product, including the 3526 bp ORF encoding DcNMCP1 (1164 amino acids; sequence data in DDBJ/EMBL-Bank/GenBank under accession number D64087) with an N-terminal 6× His tag, was digested with *Sal*I and ligated to the 5752 bp fragment from the plasmid. The fragment was amplified by PCR using the primers 5′-GCATCACCATCACCATCACGGTATG-3′ and 5′-CCCATGGTATATCTCCTTCTTAAAG-3′. The blunt ends were ligated to circularize the amplified product, which was cloned into DH5α cells (BioDynamics Lab Inc., Tokyo, Japan). After the DNA sequence was checked, a plasmid with the expected sequence, designated as pJE16N, was isolated and used for production of recombinant protein in *E. coli* strains.

A cloning site for the gene encoding DcNMCP2 with a 6× His tag was obtained by digesting pET6×HN-C with *Nco*I and *Eco*RI. The longest ORF of *DcNMCP2* from a cDNA clone (sequence data in DDBJ/EMBL-Bank/GenBank under accession number AB514509) was amplified from the cDNA clone pD2m by PCR using the primers 5′-ATGGCGAGTCCTCGATTAAC-3′ and 5′-TTCTTACTGCTCCGTCAAACTAG-3′. Subsequently, the DNA fragments were amplified by PCR using the primers 5′-CATCACCATCACCATCACGGTATGGCGAGTCCTCGATTAAC-3′ and 5′-CCAGAATTCTTACTGCTCCGTCAAACTAG-3′, resulting in a 5′-extension encoding 6× His and a 3′-extension, including an *Eco*RI restriction site. The PCR product, which included the 2808 bp ORF encoding DcNMCP2 with an N-terminal 6× His tag, was digested with *Eco*RI and ligated to the 5729 bp fragment from the plasmid. The linked fragment was amplified by PCR using DNA polymerase (KOD-Plus, Toyobo) and the primers 5′-GCATCACCATCACCATCACGGTATG-3′ and 5′-CCCATGGTATATCTCCTTCTTAAAG-3′. The blunt ends were ligated to circularize the amplified product, which was then cloned into DH5α cells (BioDynamics). Following DNA sequence analysis, a clone with the expected DNA sequence, designated as pDC2mN, was used for production of recombinant protein. The pJE16N and pDC2mN vectors were subcloned into the *E. coli* strain Rosetta II (DE3; Novagen-Merck Millipore, Darmstadt, Germany) to produce the recombinant proteins. For all PCR amplifications, high-fidelity DNA polymerase (KOD-Plus; Toyobo, Osaka, Japan) was used.

### Yield, extraction, and purification of the recombinant proteins

Overnight cultures of the *E. coli* strains were subcultured at 1:20 in 600 ml Luria broth and grown to the late exponential growth phase at 37 °C. Recombinant protein expression was induced by adding isopropyl-1-thio-β-d-galactopyranoside (Sigma-Aldrich) to a final concentration of 0.4 mM, followed by incubation for an additional 6–8 h. Next, the cells were harvested, resuspended in 20 ml Tris-buffered saline, and frozen at –80 °C. After thawing, the suspension was mixed with 80–120 ml lysis buffer consisting of Tris–HCl (pH 7.8), 100 mM NaCl, 1 mM MgCl_2_, 10 mM 2-mercaptoethanol, 0.2 mg ml^–1^ lysozyme, 0.2% Triton X-100, 25 units ml^–1^ TurboNuclease (Accelagen, San Diego, CA, USA), and a protease inhibitor mixture (Nacalai Tesque). The mixture was incubated for 20–30 min at room temperature with occasional vortexing and then centrifuged at 24 000 *g* for 10 min at 4 °C. The supernatant was collected and adjusted to 7 M urea by dissolving the solid reagent. The 6× His-tagged DcNMCP1 and DcNMCP2 proteins were purified using an IMAC resin column (Bio-Rad) with stepwise elution with a 5–250 mM imidazole gradient in 6 M urea, 50 mM Na phosphate (pH 7.8).

The purity of each recombinant protein was estimated using PAGE and western blot analyses. Each recombinant protein of interest was retained in the column with 10 mM imidazole and eluted with 125 mM imidazole. The fractions were pooled and dialysed against 8 M urea and then concentrated using an ultrafiltration device (Amicon Ultra, 30 kDa cut-off; Millipore, Burlington, MA, USA). Purified His-tagged DcNMCP1 and DcNMCP2 were lyophilized and stored at –80 °C until use.

### PAGE and immunoblotting

For PAGE, proteins were separated on NuPAGE gels containing a 4–12% polyacrylamide gradient (4–12% Bis-Tris gel; Novex-Thermo Scientific, Waltham, MA, USA) by using an MES buffer system (Novex-Thermo Scientific), according to the manufacturer’s instructions. The gels were stained with Coomassie Brilliant Blue R-250 and destained with 5% acetic acid. For immunoblotting, the proteins were electrophoretically transferred to a polyvinylidene fluoride membrane (FluoroTrans W; Pall, New York, USA) in a wet/tank blotting assembly. After transfer, the membrane was treated with a blocking solution containing 2.0% BSA (Roche) and 2.0% casein-derived blocking reagent (BlockAce; WakenBtech, Kyoto, Japan) in Tris-buffered saline for 2 h. The primary antibodies, mAbCML-1 and mAbCML-10, which were previously purified on protein G Sepharose 4 B (GE Healthcare, Chicago, IL, USA), were used at a dilution of 1:250–1:1000. For the secondary antibody, alkaline phosphatase-conjugated rabbit anti-mouse IgG (Sigma-Aldrich) was used. Phosphatase activity on the membrane was visualized using a SIGMAFAST Fast Red TR/Naphthol AS-MX Tablet (Sigma-Aldrich) staining kit.

### Protein refolding, dimerization, and assembly into higher-order structures

Purified His-tagged DcNMCP1 and DcNMCP2 were dialysed against H_2_O for 30–120 min by using a dialysis assembly (Slide-A-Lyzer MINI Dialysis Unit; Thermo Scientific) to induce protein refolding. The protein samples were then dialysed against 20 mM Na acetate buffer (pH 5.6) with or without 40 mM KCl or 40 mM Na phosphate buffer (pH 6.4) for 1–16 h at 4 °C to promote polymerization. The sample solutions were diluted 5–20 times with H_2_O before microscopy analyses and desalted using an ultrafiltration assembly, if necessary.

### Rotary shadowing, negative staining, and electron microscopy

Rotary shadowing was applied to the protein samples by mixing a 15 µl aliquot of each protein solution after dialysis with glycerol (final concentration 40%) and spraying on to freshly cleaved mica sheets. The mica was dried *in vacuo* at room temperature for at least 1 h. Next, the dried sample was shadowed on a rotating platform with a platinum–carbon projection at an elevation angle of approximately 5° by using a freeze-fracture apparatus with an ion beam sputtering system (JFD-9010; JEOL, Akishima, Japan).

For negative staining, a 10 µl aliquot of the protein sample was adsorbed on to a glow-discharged carbon-coated formvar film on a metal grid. The grid was rinsed by touching it a few times to the surface of a drop of H_2_O before it was placed on a drop of 0.75% uranyl formate (pH 4.3) for approximately 10 s. Excess liquid was blotted with a filter paper strip applied to the edge of the grid, and the grid was air-dried.

Specimens were examined under a JEM-2100 transmission electron microscope (JEOL) operated at 80 kV, and images were recorded using a charge-coupled device (CCD) camera (VELETA, Olympus Soft Imaging Solutions, Münster, Germany).

### Wide-field light microscopy

An epifluorescence microscope equipped with differential interference contrast optics (BX-50; Olympus, Tokyo, Japan) was used for conventional microscopy. MPLFLN 40×/NA 0.75 or 100×/NA 0.9 objectives with an oil immersion lens were routinely employed. Images were captured using a cooled CCD camera (C4742; Hamamatsu Photonics, Hamamatsu, Japan) driven by Aquacosmos v. 2.0 software (Hamamatsu Photonics).

### Construction of illustrations and panels

Images were processed using ImageJ v.1.46 (National Institutes of Health), Canvas Draw v.6.0 (ACD Systems, Victoria, Canada), and Canvas X 2005 (Deneba Software, Fort Lauderdale, FL, USA) software.

## Results

### STED immunofluorescence microscopy of AG3 cells

We selected the cell line AG3, which was previously established from an *A. graveolens* explant (Kimura *et al.*, 2010), as the plant material for STED microscopy. The high optical transparency and low background fluorescence of AG3 cells are well suited for imaging using whole-cell specimens. The nuclear lamina of AG3 cells was marked by specifically staining NMCP1 and NMCP2 using antibodies that were previously generated against carrot nuclear matrix proteins (Masuda *et al.*, 1993) (Supplementary Fig. S1A).

First, we examined the differences between STED microscopy and LSM with regard to the resolution. Images focused on an identical plane of a field showed the difference in resolution between microscopes with the respective optical modes (Supplementary Fig. S1B). The planar resolution of STED microscopy was affected not by the relative intensity of the xy-STED laser, but by the relative intensity of the z-STED laser and time-gates that were applied after each excitation laser pulse to gate out the STED peak. A resolution of <30 nm was estimated from actual images obtained using a STED microscope after adjusting the settings (Supplementary Fig. S1B). This resolution was comparable with previously reported values (Schoonderwoert *et al.*, 2013; Wegel *et al.*, 2016) and was nearly six times higher than the theoretical resolution of light microscopy. Serial z-stacks adjusted to 100 nm steps yielded completely resolved images (Supplementary Fig. S1C). From this distance, we estimated the z-axis resolution to be >100 nm.

In this study, immunofluorescence signals with especially low intensities were visualized by usually equalizing the signal intensity histograms by applying gamma correction. In addition to the reduction of available photons for each pixel owing to the magnification, the application of the second laser pulse quenched the photons in STED microscopy. We applied automatic noise reduction, signal restoration, and de-blurring processes (Supplementary Fig. S1B) for signal intensity histogram equalization. Histogram equalization was used to visualize the complete intensity range of the immunofluorescence signals and to image higher-order structures of NMCPs.

### Higher-order structures of AG3 cell NMCP1 and NMCP2

**Fig. 1.**
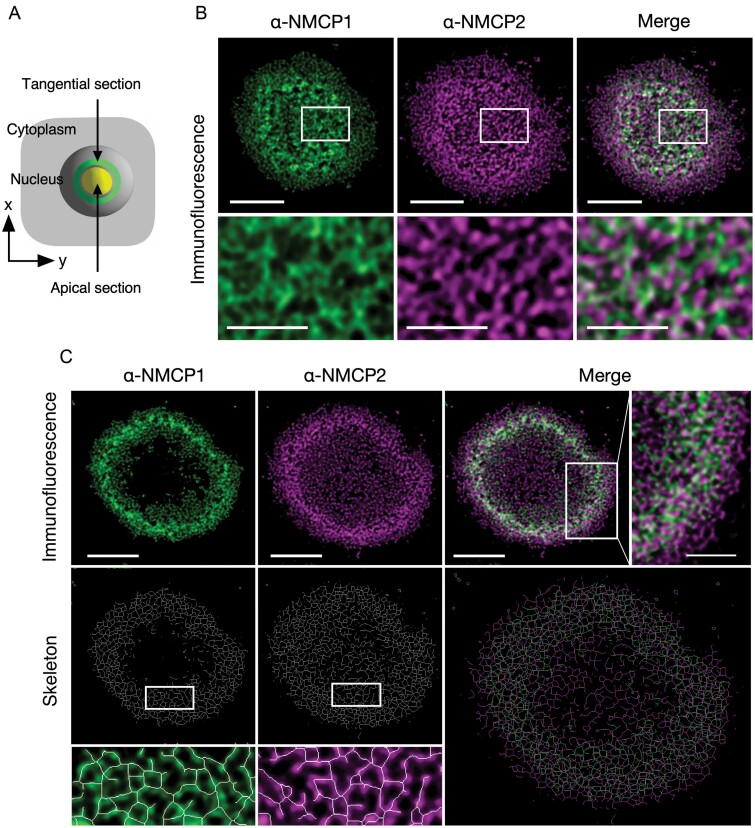
Filamentous networks made of NMCP1 and NMCP2. AG3 cells were stained with Alexa Fluor 488-labelled Fab fragments (ZENON) previously conjugated with anti-NMCP1 antibody (mAbCML-1). NMCP2 was stained using a conventional immunofluorescence method by using mAbCML-10 and Alexa Fluor 555-labelled secondary antibody. The STED laser power was adjusted to 50% of the emission laser power. The z-STED laser power was adjusted to 40% of the emission laser power. Images were captured through time-gates opened from 0.5 ns to 6.5 ns after laser pulse excitation. Immunofluorescence images from nuclear sections focused to apical and tangential planes along the z-axis were obtained. (A) Illustration representing the apical and tangential nuclear plane sections. (B) Images from a section focused to the apical nuclear plane. The boxed areas in the upper panels are magnified in the lower panels. (C) Immunofluorescence images from a section focused to the tangential plane (upper panels). The boxed area in the merge is magnified and presented in the right panel. The lower panels show skeletal elements (skeletons) extracted from immunofluorescence signals. the boxed areas are magnified in the panels underneath. The magnifications are superimposed on the greyscaled immunofluorescence image. Scale bars=2.5 µm (whole nuclear section images) and 1.0 µm (magnifications).

To characterize the *in vivo* higher-order structure of NMCP1 and NMCP2, we analysed the images obtained from three nuclear sections along the z-axis, namely, apical (the nuclear section nearest to the coverslip), tangential (approximately 1 µm away from the apical section) (Fig. 1A), and middle (the cross-section showing the maximum nuclear area) sections. The STED images focusing on the apical (Fig. 1B) and tangential (Fig. 1C) sections revealed the assembly of NMCP1 and NMCP2 into filamentous networks extending across the nuclear periphery. The individual filaments were more clearly observed in the oblique NL optically cutting against the axis in the tangential sections than in the NL image parallel to the nuclear surface in the apical sections.

To characterize the networks composed of NMCP1 or NMCP2 filaments, we simplified the immunofluorescence image to single-pixel-width elements (Supplementary Fig. S2) by using tools built into ImageJ software. The automatically generated skeletons indicated that NMCP1 and NMCP2 formed linear filaments with branches connecting with each other, and formed distinct networks consisting of the respective proteins (Fig. 1C). The opening size (the size of the face enclosed by skeletonized segments) in the NMCP1-derived skeleton was 0.053±0.031 µm^2^, and that in the NMCP2-derived skeleton was 0.042±0.027 µm^2^. Thus, the openings did not show a significant difference in size.

### Characterization of the NMCP-based nuclear lamina

**Fig. 2.**
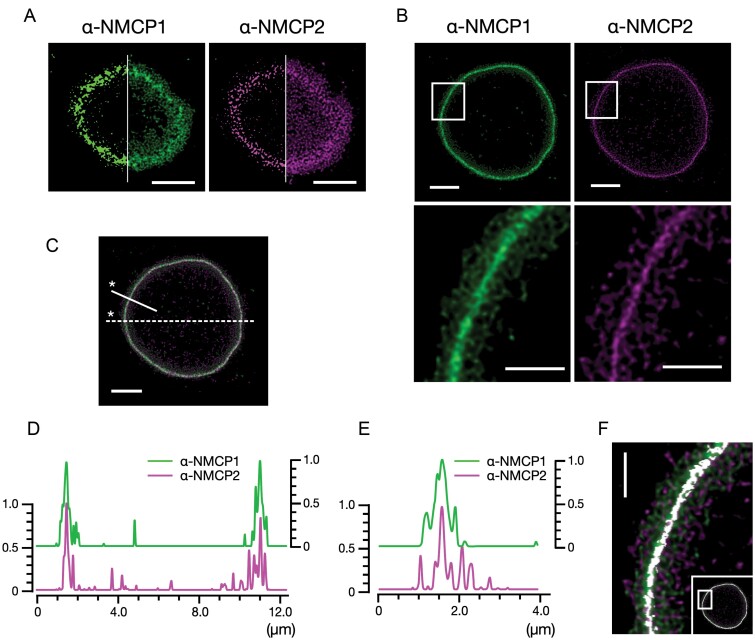
Localization of thick NMCP filaments at the nuclear lamina. (A) Distributions of NMCP1 and NMCP2, showing exclusive localization of thick filaments at the inner zone of the network. The left half of each image shows a binarized image generated by extracting 255 scales from Alexa Fluor 488 and Alexa Fluor 555 signals by setting the upper and lower threshold values to 120 and 80, respectively. The original immunofluorescence image data are the same as those in Fig. 1C. (B) Images from a section focused to the middle nuclear plane, showing extensive localization of NMCP1 and NMCP2 at the nuclear lamina. Immunofluorescence staining of the cells and STED microscopy were performed in the same manner as those for imaging apical and tangential plane sections, except for the focal plane. The boxed areas in the upper panels are magnified in the lower panels. (C) A merged picture generated from the colour channels presented in (B). The solid and dashed lines indicate the selection for line plots. The asterisks show the starting points. (D) Line plots on the dashed line indicated in (C). Ordinates, relative fluorescence intensity (arbitrary units); abscissa, distance. (E) Line plots on the solid line indicated in (C). Ordinates, relative fluorescence intensity (arbitrary units); abscissa, distance. (F) Overlaps between the distributions of NMCP1 and NMCP2. Overlapping areas were selected and extracted from the merged image data by adjusting thresholds in the HSB colour space. Overlapping areas are shown in white. The main image is a magnification of the boxed area shown in the inset. Scale bars=2.5 µm (whole nuclear section images) and 1.0 µm (magnifications).

The NL has been characterized by a dense layer composed of filamentous proteins, lamins. We examined the architecture that characterizes the high-density distribution of lamina proteins by using immunofluorescence images obtained from a tangential plane section. High-intensity signal fractions extracted over an intensity threshold highlighted the thick segments distributed within the networks (Fig. 2A). The thick segments were localized in the inner zone of the network. The inner zone corresponding to the NL appeared on the images from a middle nuclear section (Fig. 2B). A middle nuclear section, which represented a typical image of the NL extending across the nuclear periphery (Fig. 2B), hardly showed the filamentous assembly because of the dense distribution of NMCP1 and NMCP2 at the NL.

Line plot analyses of the immunofluorescence intensity across the entire nuclear section (Fig. 2C, D) and a narrow range around the nuclear periphery (Fig. 2C, E) showed a predominant localization of NMCP1 and NMCP2 at the NL. The application of a program for the extraction of the merged colour channel from the immunofluorescence datasets (Fig. 2F) showed that NMCP1 and NMCP2 did not layer, suggesting that these components formed a single-layered filamentous lamina.

### Close associations between NMCP1 and NMCP2 filaments

**Fig. 3.**
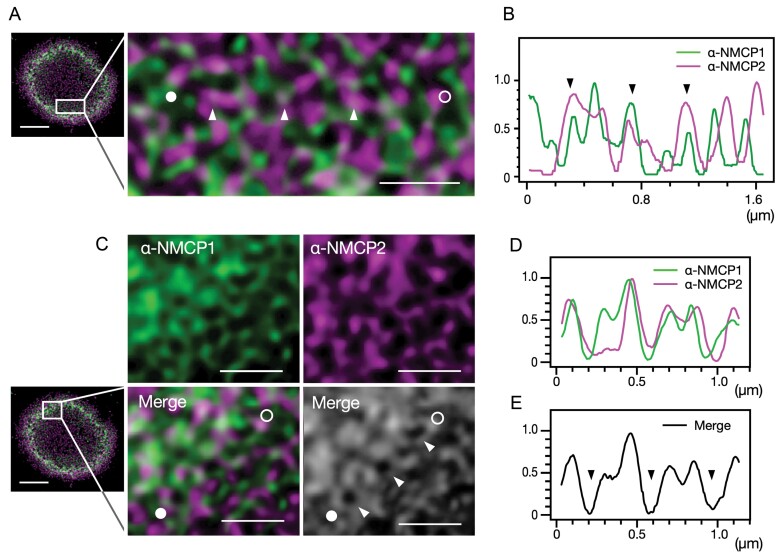
Associations between NMCP1 and NMCP2 filaments. (A) Close associations between NMCP1 and NMCP2 filaments (arrowheads). In the enlarged image (right), a virtual line from the solid to the open circle indicates the line selection for the line plot analysis. (B) Line plots along the virtual line indicated in (A). Arrowheads indicate overlaps of intense signals from both colour channels, showing a close association between NMCP1 and NMCP2 filaments. Arrowheads correspond to those in (A). (C) Associations between NMCP1 and NMCP2 filaments at the perimeter of NMCP-depleted holes. Virtual lines from the solid to the open circles in the lower panels indicate the line selections used for line plots. (D) Line plots for the green and magenta channels along the line indicated in the colour merged image in (C). (E) Line plots along the virtual line indicated in the greyscale image in (C). Arrowheads correspond to the NMCP-depleted holes indicated in the greyscale image in (C). Scale bars=2.5 µm (whole nuclear section images) and 0.5 µm (magnifications).

STED imaging with nanometre-scale resolution allowed the discrimination of close molecular associations between the NMCP1 and NMCP2 filaments. The associations showed a colour shift caused by the two merged colour channels (Fig. 3A) and were confirmed on the line plots as overlapping intensity peaks (Fig. 3B). The distance between both filaments at contiguous points was estimated to be <100 nm from the resolution along the optical z-axis.

NMCP1 and NMCP2 filaments frequently run to enclose an almost circular-shaped NMCP-depleted hole with a diameter of approximately 150–190 nm (Fig. 3C). The colour shifts were notably detected at the perimeter of the NMCP-depleted holes. Comparing the intensity peaks along line plots indicated that close associations between NMCP1 and NMCP2 filaments occurred at the perimeter of NMCP-depleted holes (Fig. 3D, E).

### Overlap between the NL and chromatin distribution

**Fig. 4.**
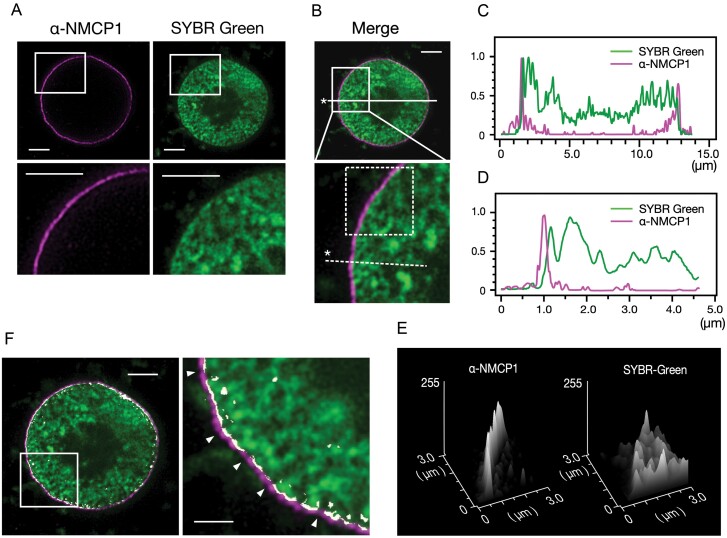
Overlap between chromatin and NMCP1 distributions. (A) An AG3 cell nucleus stained with Alexa Fluor 555-linked Fab fragments (ZENON) conjugated with an anti-NMCP1 antibody (mAbCML-1) (left) and SYBR Green I (right). The section was focused on the middle plane of the nucleus and imaged under conditions similar to those used to produce Fig. 2B, except that the STED laser output was adjusted to 40% of the emission laser power. The lower panels are magnifications of the boxed areas in the upper panels. (B) A merge of the images presented in (A). The area enclosed by the solid box in the upper panel is shown magnified in the lower panel. The solid and dashed lines indicate the selections for the line plots presented in (C) and (D). The asterisks indicate the starting points for the plots. The area enclosed by the dashed box shows the selection for the surface plots presented in (E). (C) Line plots along the solid line in (B). Ordinate, relative fluorescence intensity (arbitrary units); abscissa, distance. (D) Line plots along the dashed line in (B). Ordinate, relative fluorescence intensity (arbitrary units); abscissa, distance. (E) Surface plots of the area enclosed by a dashed box in (B). (F) Overlap between NMCP1 and DNA distributions. The area enclosed by the box in the left panel is shown magnified in the right panel. The overlap was selected by adjusting colour thresholds in the HSB colour space (shown in white). Arrowheads show chromatin invaginations. Scale bars=2.5 µm (A, B, whole nuclear section in F) and 0.5 µm (magnification in F).

The image from a middle nuclear section after immunofluorescence staining for NMCP1 combined with DNA staining to visualize chromatin revealed that the NL extended across the periphery of the nucleoplasm (Fig. 4A, B). The line plots along lines traversing the nucleus (Fig. 4B, C) and a narrow nuclear section (Fig. 4B, D) applied to the same image showed that the NL extended across the periphery of the nucleoplasm. Detailed line plots around the nuclear periphery revealed that the outermost DNA or chromatin distribution overlapped with the nucleoplasm-facing region of the NL (Fig. 4D). The surface plot analysis extended the data equivalent to those obtained from the line plots (Fig. 4D) to an area including the NL (Fig. 4E). AG3 cells did not show predominant condensed chromatin localization at the nuclear periphery.

DNA staining revealed invaginations of chromatin in places along the nucleoplasm surface (Fig. 4F). The immunofluorescence image of NMCP1 distribution in the middle nuclear section was discontinuous and showed gaps at places where the chromatin was invaginated. Imaging of the spatial overlap of the chromatin and NMCP1 distributions by adjusting the colour threshold revealed that the formation of NL gaps was involved with chromatin invaginations (Fig. 4F).

**Fig. 5.**
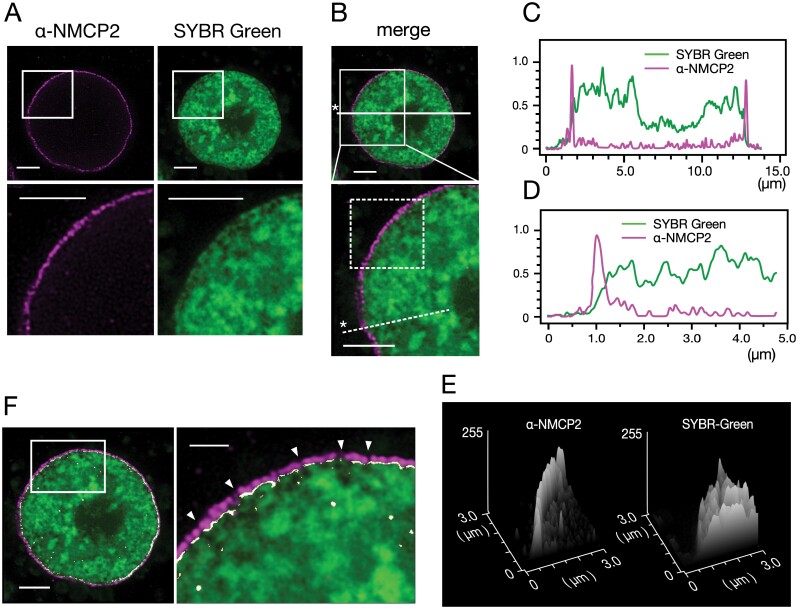
Overlap between chromatin and NMCP2 distributions. (A) An AG3 cell nucleus stained with Alexa Fluor 555-linked Fab fragments (ZENON) conjugated with an anti-NMCP2 antibody (mAbCML-10) (left) and SYBR Green I (right). The section was focused on the middle plane of the nucleus and imaged under conditions similar to those used to produce Fig. 2B, except that STED laser output was adjusted to 40% of the excitation laser power. The lower panels are magnifications of the boxed areas in the upper panels. (B) A merge of the images presented in (A). The area enclosed by the solid box in the upper panel is shown magnified in the lower panel. The solid and dashed lines indicate the selections for the line plots presented in (C) and (D). The asterisk indicates the starting point for the plots. The area enclosed by the dashed box shows the selection for the surface plots presented in (E). (C) Line plots along the solid line in (B). Ordinate, relative fluorescence intensity (arbitrary units); abscissa, distance. (D) Line plots along the dashed line in (B). Ordinate, relative fluorescence intensity (arbitrary units); abscissa, distance. (E) Surface plots of the area enclosed by a dashed box in (B). (F) Overlap between NMCP2 and DNA distributions. The area enclosed by the box in the left panel is shown magnified in the right panel. The overlap was selected by adjusting colour (hue) and brightness thresholds in the HSB colour space (shown in white). Arrowheads show chromatin invaginations. Scale bars=2.5 µm (A, B, whole nuclear section in F) and 1.0 µm (magnification in F).

After staining for NMCP2 and chromatin, images from the middle nuclear section revealed an overlap between NL and chromatin (Fig. 5A, B). Data from the line plots (Fig. 5C, D) and surface plot analyses (Fig. 5E) support the spatial relationship between the NL and chromatin. Gaps in the NL involved with chromatin invaginations were imaged using a methodology similar to that applied to analyse the overlaps of the NMCP1 and DNA distributions (Fig. 5F).

### Assembly of His-tagged DcNMCP1 and DcNMCP2 into filaments

**Fig. 6.**
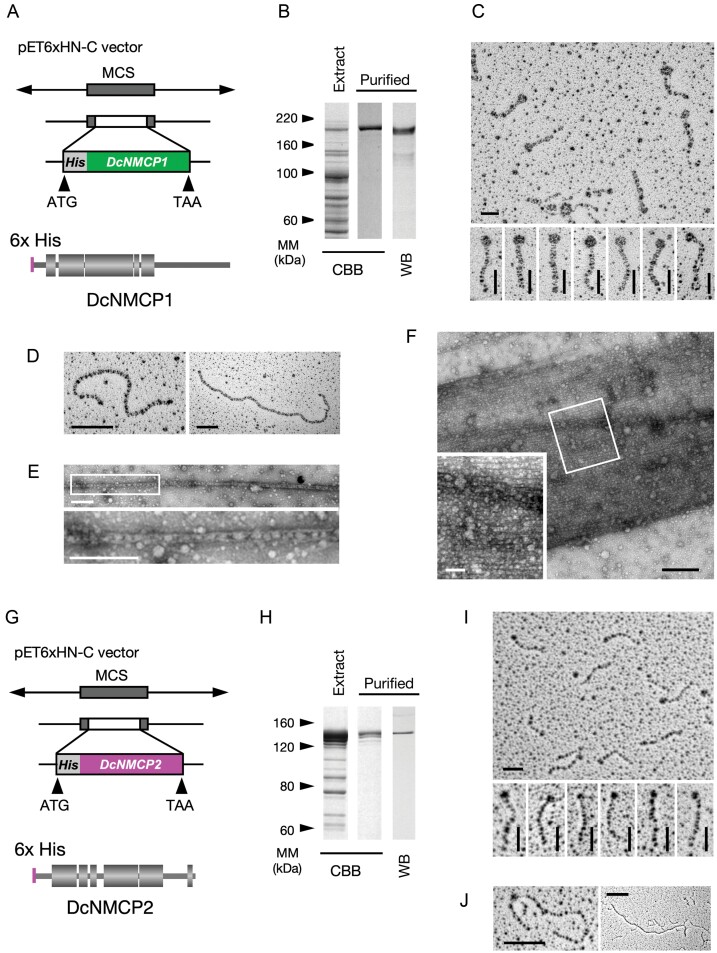
Assembly of His-tagged DcNMCP1 and DcNMCP2 into filaments. (A) A vector constructed for 6× His-tagged DcNMCP1 expression in recombinant *E. coli* (upper diagram) and a predicted structure of His-tagged DcNMCP1 (lower diagram). MCS, multiple cloning site. (B) NuPAGE gel electrophoresis (CBB, Coomassie Brilliant Blue) and western blot (WB) imaging of His-tagged DcNMCP1. Affinity-purified 6× His-tagged DcNMCP1 was dissolved in 7 M urea and dialysed against H_2_O for 60 min to induce refolding, and then against 20 mM Na acetate (pH 5.6) with or without 40 mM KCl to enhance polymerization. Protein samples were subjected to rotary shadowing or negative staining for transmission electron microscopy. (C) Coiled-coil dimers imaged under an electron microscope after low-angle metal shadowing. Scale bars=50 nm. (D; E) Filaments formed after dialysis against 20 mM Na acetate (pH 5.6) for 2 h. Protein samples were contrasted by low-angle rotary shadowing (D) or by negative staining (E). Bars=200 nm. (F) A filament bundle formed after dialysis against 20 mM Na acetate containing 40 mM KCl for 4 h, showing lateral associations of filaments. The protein sample was imaged by negative-staining electron microscopy. Scale bars=500 nm and (inset) 100 nm. (G) A vector constructed for 6× His-tagged DcNMCP2 expression in recombinant *E. coli* (upper diagram) and a predicted structure of His-tagged DcNMCP2 (lower diagram). (H) NuPAGE gel electrophoresis (CBB) and WB imaging of His-tagged DcNMCP2. Affinity-purified 6× His-tagged DcNMCP2 was prepared for electron microscopy as described above for DcNMCP1. (I) Coiled-coil dimers imaged under an electron microscope after low-angle rotary shadowing. Bars=50 nm. (J) Filaments formed after dialysis against 20 mM Na acetate (pH 5.6) with 40 mM KCl for 6 h. Bars=200 nm.

Considering that self-assembly is an intrinsic and key propensity of lamins (Goldman *et al.*, 2002), we examined NMCP1 and NMCP2 assembly into filaments by using His-tagged DcNMCP1 (Fig. 6A) and DcNMCP2 (Fig. 6G) expressed in *E. coli*. The recombinant proteins produced in *E. coli* were present in the insoluble fraction under a wide range of culture conditions for the expression. Therefore, the proteins were dissolved in 6–8 M urea, purified in the presence of urea, and subjected to molecular refolding. Purification was performed by DEAE ion-exchange chromatography, followed by affinity chromatography using a tag-specific binding resin. Electrophoresis of the proteins showed a major band corresponding to His-tagged DcNMCP1 (Fig. 6B) and DcNMCP2 (Fig. 6H).

Purified His-tagged DcNMCP1 and DcNMCP2 refolded and dimerized after dialysis against H_2_O (Fig. 6C, I). Electron micrographs of the metal-shadowed protein samples showed rod-shaped proteins with a globular part at one end. The His-tagged DcNMCP1 dimers had a large globule at one end (Fig. 6C), whereas the His-tagged DcNMCP2 dimers had a small globule (Fig. 6I). We predicted that these globules corresponded to the CTD. Although measurements of these globular proteins on electron micrographs after metal shadowing were not quantitative, the relative sizes were consistent with the difference in the sequence lengths of their CTDs. The lengths of the rods in His-tagged DcNMCP1 and DcNMCP2 dimers were 93.6±7.8 nm and 100.1±9.9 nm, respectively (Supplementary Fig. S3A, B). These lengths were almost consistent with the rod lengths estimated from the linear relationship based on amino acid numbers and rod length in chicken lamin B (Supplementary Fig. S3C) (Heitlinger *et al.*, 1991). These relationships suggested that the rod parts consist mainly of coiled coils, and that the actual rod length in refolded proteins is conserved between NMCP1 and NMCP2 (Supplementary Fig. S3D).

Following dialysis against H_2_O for 30–60 min, protein samples were incubated in 20 mM Na acetate (pH 5.6) containing 40 mM KCl. Polymerization into filaments was promoted by adding KCl or NaCl, but at concentrations of >40 mM protein precipitates rapidly formed. Polymerization into filaments was also promoted in the presence of 40 mM Na phosphate (pH 6.4) and resulted in the formation of protein precipitates. These precipitates are unsuitable for structural analysis by using electron microscopy. In contrast, filament formation was detected in solutions containing <20 mM Na phosphate without KCl or NaCl. The filaments were imaged using transmission electron microscopy after low-angle rotary shadowing (Fig. 6D) or negative staining (Fig. 6E). The His-tagged DcNMCP1 filaments imaged using negative-staining electron microscopy showed two parallel strands at a distance of approximately 32 nm with globular structures aligned between the strands (Fig. 6E). We inferred that the strands corresponded to the rod domain, and that the globules corresponded to the CTD. With prolonged incubation, filament bundles appeared in the sample solutions (Fig. 6F). Each bundle was composed of a structure with a thickness of ~32 nm, which was structurally similar to the separately formed filaments.

These results indicate that the DcNMCP1 analogue refolded to form coiled-coil dimers, and that the dimers self-assembled into filament bundles via filament formation. The dimerization and assembly into polymers was confirmed with high reproducibility in separate experiments starting with His-tagged DcNMCP1 production in recombinant *E. coli*.

Conversely, His-tagged DcNMCP2 dimers hardly assembled into filaments under incubation conditions similar to those used for His-tagged DcNMCP1; most of the His-tagged DcNMCP2 proteins remained as dimers or oligomers of <300 nm (Fig. 6J). Filament bundles were not formed. The recalcitrance of His-tagged DcNMCP2 to self-assemble into linear filaments resulted in poor information on the filament structure and assembly of NMCP2 polymers.

## Discussion

### NMCP1and NMCP2 form filamentous networks

In this study, we showed that in AG3 cells NMCP1 and NMCP2 formed networks composed of filamentous polymers (Fig. 1B, C). We achieved this by incorporating recent technology in fluorescence microscopy to break the diffraction limit and thereby improve the resolution. The filamentous networks extended across the nuclear periphery, which confirmed that these components may play a role in scaffolding the NE and have a structurally similar assembly to the lamin-based NL of animals (Aebi *et al.*, 1986). The NL is widely accepted to be composed of orthogonally assembled lamin filaments, and these assemblies are a hallmark of lamins. This model is based on the electron microscopic analysis of amphibian oocyte nuclei. Recently, in mouse adult fibroblast tissues, a lamin assembly showing intricate filamentous networks has been reported, suggesting that the orthogonal assembly is an example that only a single major type lamin (L_III_ lamin in *Xenopus laevis* oocytes) is involved (Xie *et al.*, 2016). The existence in a plant system of an NL with assembly similar to those in animal systems suggests that the NL might have emerged to meet similar evolutionary pressures applied to plants and animals.

Possible structural associations between AG3 cell NMCP1 and NMCP2 filaments provide structural evidence for their functional relationship (Fig. 3), which has been proven for their orthologues in Arabidopsis. Mutants with a deletion of *CRWN4* express a phenotype similar to that of the *crwn1* mutant when the counterpart gene is mutated; both mutations form abnormal, spherical, and small nuclei and show an anomalous nuclear position in the cell (Sakamoto and Takagi, 2013; Wang *et al.*, 2013). Most recently, Blunt *et al.* (2020) reported that the level of CRWN4 within the nucleus is modulated by the abundance of CRWN1 or its paralogous proteins, indicating that the functional interactions between CRWN1 and CRWN4 are mediated by their molecular associations. Our structural analyses of the AG3 cell NL strongly support these findings.

### NMCP filament bundles characterize the plant NL

STED microscopy revealed that high-intensity signals from the NL were caused by the localization of thick NMCP filaments. Images from different sections helped to fully understand the assembly of the NL. By using an *in vitro* system exploiting His-tagged carrot NMCP1, we found that the thickening resulted from lateral associations between filaments. The assembly with lateral associations is reminiscent of *in vitro* self-assembly of lamins into paracrystals (Heitlinger *et al.*, 1991; Turgay *et al.*, 2017).

**Fig. 7.**
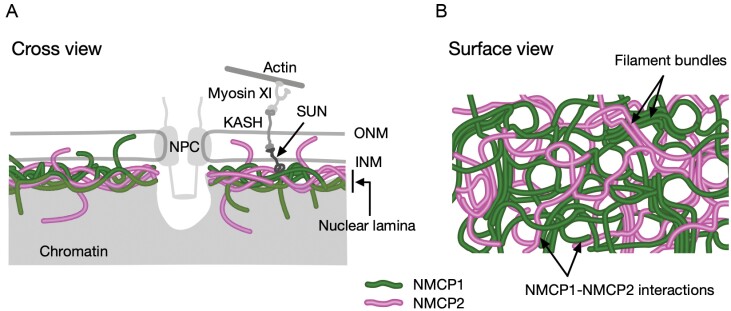
Model of the structure of the NMCP-based NL. The plant NL is a network comprising NMCP1 and NMCP2 filaments. The core region of the network contains filament bundles and forms a dense filamentous layer. The chromatin distribution overlaps with the nucleoplasm-facing region of the NL. The NL has openings, associated with chromatin invaginations, that are thought to accommodate the nuclear pore complex (NPC). (A) Cross-sectional view. (B) Surface view. INM, inner nuclear membrane; ONM, outer nuclear membrane.

Lateral associations notably decreased the solubility of NMCP1 filaments and caused precipitation from the solution. We infer that the insolubility of the polymers with lateral associations may be the cause of lamina-specific physicochemical traits, namely, their resistance to extractions with high-salt- and non-ionic detergent-containing solutions (Moreno Díaz de la Espina *et al.*, 1991; Masuda *et al.*, 1993). The Arabidopsis integral inner nuclear membrane proteins AtSUNs, which interact with KASH proteins to form a part of the LINC complex spanning the nuclear membrane and bridging between the NE and the cytoskeleton (Graumann *et al.*, 2010; Zhou and Meier, 2013), associate with CRWN1 and immobilize the lamina protein in the NE (Graumann, 2014). NMCP1 networks including insoluble filament bundles may play a role in maintaining nucleo-cytoskeletal bridges by stabilizing associations between the NL and AtSUNs (Fig. 7).

We failed to determine a reliable protocol to form filamentous polymers and filament bundles from His-tagged DcNMCP2 coiled-coil dimers. We inferred that the difference in the polymerizing ability of His-tagged DcNMCP2 compared with His-tagged DcNMCP1 may be attributed to the functions of the CTD, for the following reasons. The significant protein sequence similarity between NMCP1 and NMCP2 is restricted to the rod domain, including a narrow region near the rod domain. The CTD of NMCP2-type members lacks a sequence including the R/QHYNLRR/H motif that precedes a functional nuclear localization signal and functions to localize the protein to the nuclear periphery (Kimura *et al.*, 2014) (Supplementary Fig. S4). This region is thought to be critical for NMCP1 to form filaments and assemble into the NL. Lamins include a highly conserved region, the immunoglobulin (Ig) fold domain, in the C-terminal domain, which plays a core role in assembly into head-to-tail associations (Izumi *et al.*, 2000; Shumaker *et al.*, 2005; Prokocimer *et al.*, 2009). The filament assembly imaged using negative-staining electron microscopy suggested that the CTD of NMCP1 may play a significant role in the association between NMCP1 dimers (Fig. 6, Supplementary Fig. S3). The recalcitrance of His-tagged DcNMCP2 to self-assemble into oligomers raises the possibility that interactions with NMCP1 might be required to stabilize the NMCP2 filaments in the nucleus.

Immunofluorescence data from the middle nuclear sections showed less organized filaments extending around the NL (Fig. 2B, Supplementary Fig. S1). We infer that some NMCP1 and NMCP2 filaments remain unorganized at the end of mitosis, and that the organization of the NL continues to occur in interphase, associated with the growth of the NE.

Filament thickening occurred within the network to form a single-layered NL network composed of NMCP1 and NMCP2. The assembly without layers is thought to be caused by close associations between both types of filaments. This may be contrasted with an example of A/C-type and B-type lamin layers within the NL that perform distinct roles (Nmezi *et al.*, 2019).

### Peripheral chromatin colocalizes with the NL

Recent Hi-C analysis data from Arabidopsis have revealed a non-random domain organization of chromatin at the nuclear periphery (Bi *et al.*, 2017). Furthermore, Arabidopsis CRWN1 has been shown to mediate chromatin tethering to the NE and to act as a key modulator of chromatin modifications at the nuclear periphery (Hu *et al.*, 2019; Mikulski *et al.*, 2019). Most recently, the interaction between CRWN1 and the copper-associated gene locus has been reported (Sakamoto *et al.*, 2020). In this case, CRWN1 likely interacts with chromatin or directly with the genes that function at the nuclear periphery (Sakamoto *et al.*, 2020). To establish a structural basis for these advances in the interaction between the NL and chromatin, we examined the chromatin distribution at the nuclear periphery and its spatial relationships with the NL. The spatial overlap between chromatin and the NL around their boundaries is consistent with the findings of previous studies on chromatin tethering to CRWN1, providing a structural basis for chromatin organization at the nuclear periphery.

Examination of the middle sections along the z-axis of a nucleus revealed cavities caused by nucleoplasm invaginations existing in places across the nuclear periphery. The hole enclosed by the NMCP filaments associated with chromatin invaginations is thought to be a structure for accommodating the nuclear pore complex (NPC). Associations between NMCP1 and NMCP2 filaments enclosing the NMCP-depleted holes suggest that these proteins coordinate to form a framework for the chromatin invaginations.

Localization of highly condensed chromatin clusters to the nuclear periphery was not observed in AG3 cells. SRM imaging of chromatin fractions stained with molecular markers could help to characterize interactions between the NL and peripheral chromatin and to unravel chromatin organization at the nuclear periphery.

### Structure model for the NMCP-based NL

A hypothetical model for the plant NL, including new experimental evidence obtained mainly from SRM analysis of the AG3 cell NL, is shown in Fig. 7. The illustration depicts networks composed of NMCP1 and NMCP2 filaments. The filaments partially form bundles extending across the nuclear periphery, which characterize the NL. This model is based only on the analysis of an NL of actively dividing cells and therefore reflects the nuclear dynamics in meristem cells.

### Conclusion

We identified highly organized filamentous networks with filament bundles extending across the nuclear periphery in a plant cell system. The existence of the filamentous lamina might suggest that the NMCP-based NL plays a role analogous to that of the lamin-based NL, and that both NLs emerged under common evolutionary pressures. Conversely, structural differences between the NMCP- and the lamin-based NL are compatible with the recent hypothesis that NMCPs emerged and evolved independently of the lamin lineage. In this study, together with NMCP1, NMCP2 was shown to be a major component of the NL; it constitutes a moiety of the interconnected filamentous networks. The overlaps between the distributions of NMCP1, NMCP2, and chromatin confirmed functional associations of the NL with chromatin. The super-resolution imaging of the NL components at the protein level could make a significant contribution to the elucidation of NE functions and might impact debates on the evolutionary history of the plant NL.

## Supplementary data

The following supplementary data are available at *JXB* online.

Fig. S1. Comparison of imaging by wide-field microscopy, CLSM, and STED microscopy.

Fig. S2. Extraction of skeletal elements from immunofluorescence microscopy images.

Fig. S3. Structure models for DcNMCP1 and DcNMCP2 dimers.

Fig. S4. Protein motifs of the C-terminal domain of DcNMCP1 and DcNMCP2.

erab243_suppl_Supplementary_Figures_S1-S4

## Data Availability

All data supporting the findings of this study are available within the paper and within its supplementary materials published online.

## Abbreviations

CTD: carboxy-terminal domain
HSB: hue–saturation–brightness
IF: intermediate filament
NE: nuclear envelope
NL: nuclear lamina
NPC: nuclear pore complex
SRM: super-resolution microscopy
STED: stimulated emission depletion
