# Supplementary material for: The plant nuclear lamina proteins NMCP1 and NMCP2 form a filamentous network with lateral filament associations
Source: J Exp Bot. 2021 Jun 4;72(18):6190–204. doi: 10.1093/jxb/erab243 (PMC8483785; doi:10.1093/jxb/erab243)
Supplement: erab243_suppl_Supplementary_Figures_S1-S4 [file erab243_suppl_supplementary_figures_s1-s4.pdf]

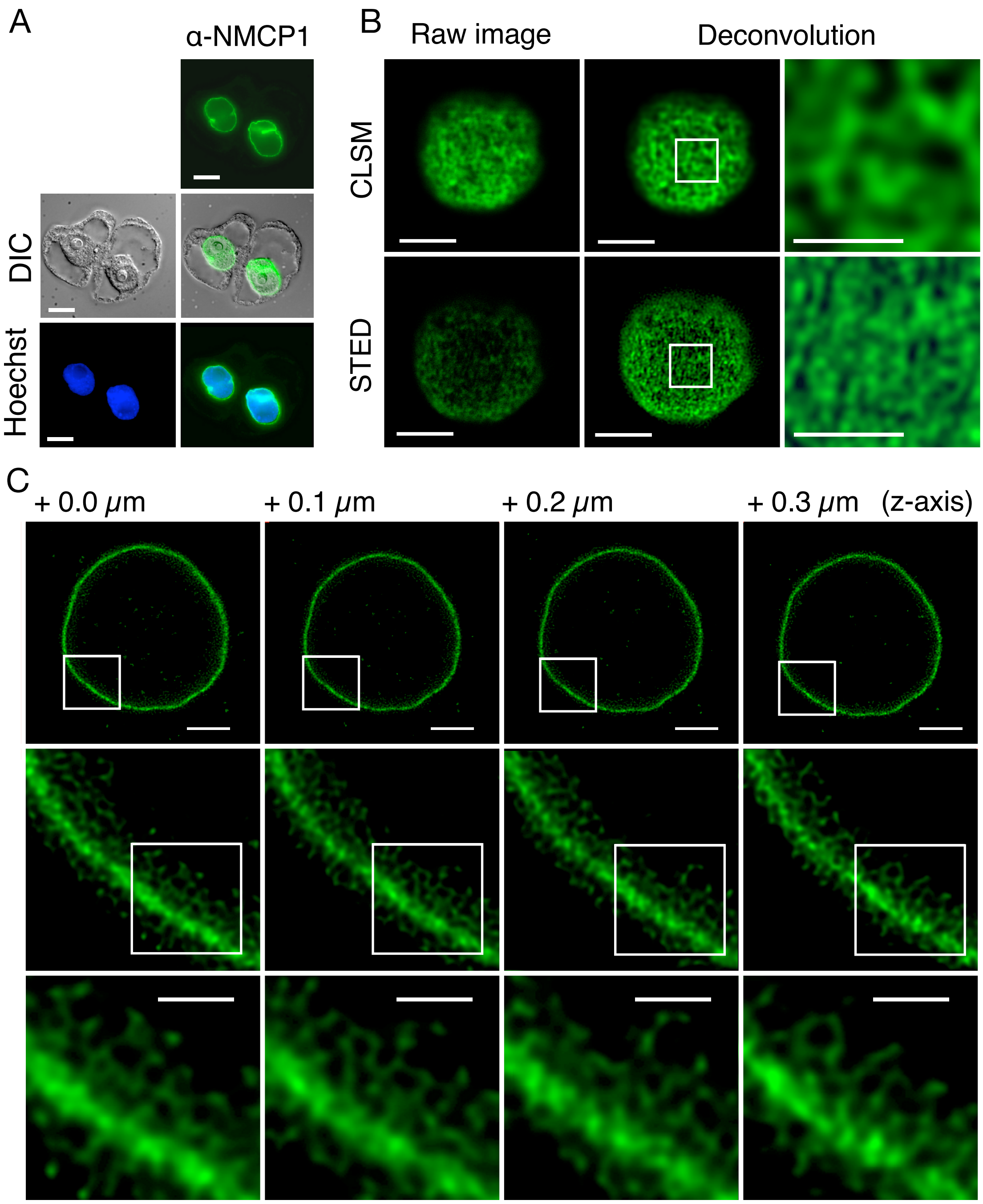

Fig. S1. Comparison of imaging by wide field microscopy, CLSM, and STED microscopy. AG3 cells were immunostained with an NMCP1-specific antibody (mAbCML-1) coupled with Alexa Fluor 488-conjugated Fab fragments (ZENON). (A) NMCP1 localisation at the nuclear periphery revealed by wide field microscopy; nuclei were counter-stained with Hoechst 33258. Scale bars, 10 μm. (B) Comparison between conventional confocal laser scanning microscopy and STED microscopy; images taken from the same field at an apical plane section of the nucleus. Scale bars, 2.5 μm and 0.5 μm in magnifications (rightmost panel). (C) Stacks of middle nuclear sections, captured at 100 nm intervals along the z-axis. Areas enclosed by rectangles are magnified below the respective images. Scale bars, 2.5 μm (top row); 0.5 μm (bottom row).

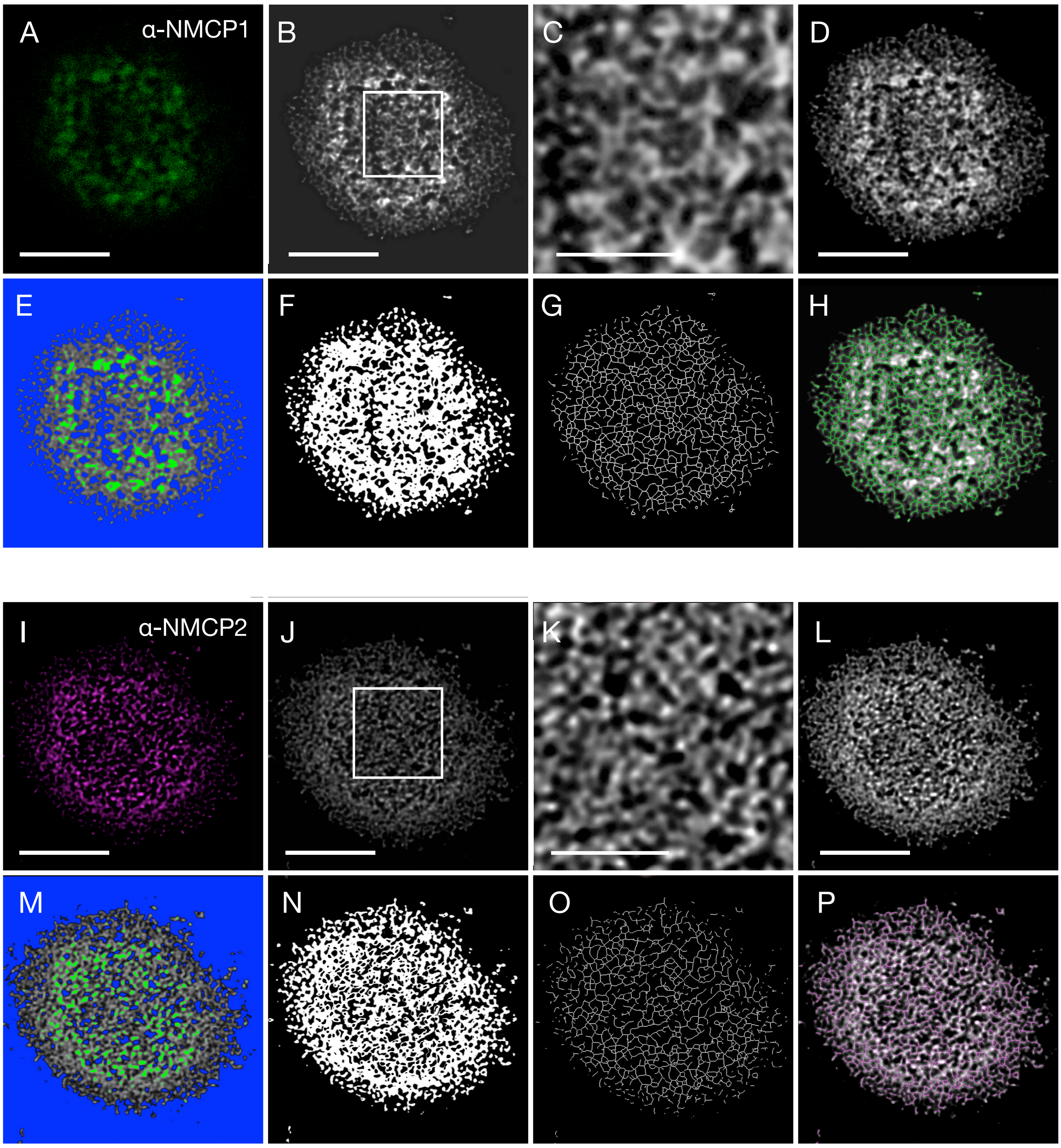

Fig. S2. Extraction of skeletal elements from immunofluorescence microscopy images. (A)–(H) Nuclear skeletal element extraction from a STED immunofluorescence image of AG3 cells labelled using anti-NMCP1 antibody (mAbCLM-1). (I) –(P) Nuclear skeletal element extraction from a STED immunofluorescence image of AG3 cells labelled using anti-NMCP2 antibody (mAbCLM-10). (A) and (I) Raw STED images. (B) and (J) STED images after the application of a deconvolution algorithm. (C) and (K) Magnification of areas enclosed by rectangles in (B) and (J). (D) and (L) Images after the application of γ-correction to grey scale to correct the contrast. (E) and (M) Images after adjustment of upper/lower thresholds to exclude extremely intense signals (shown in green) and background noise (shown in blue). (F) and (N) Images after conversion to binary. (G) and (O) Skeletonisation. (H) and (P) Skeletons superimposed on immunofluorescence images. Scales, 1.0 μm in (C) and (K); 2.5 μm (other images).

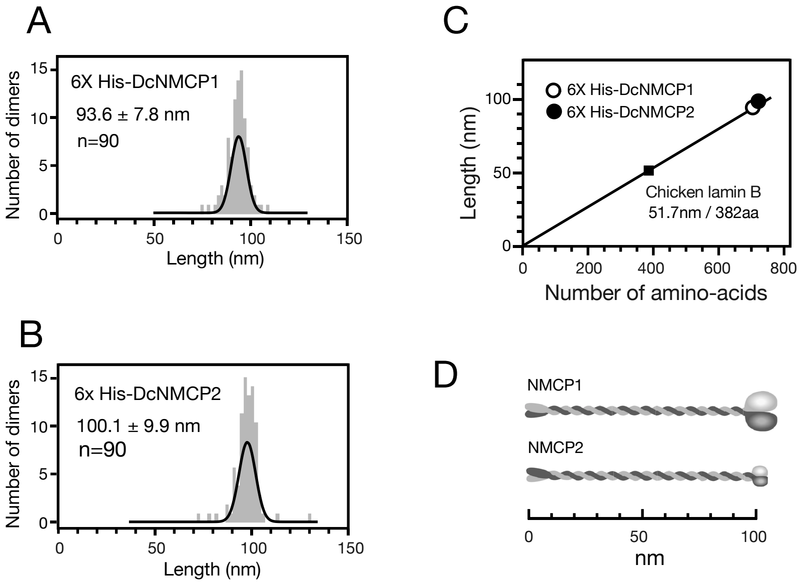

Fig. S3. Structure models for DcNMCP1 and DcNMCP2 dimers. (A) and (B) The rod length distribution of 6× His-tagged DcNMCP1 (A) and 6× His-tagged DcNMCP2 (B) dimers, measured on electron micrographs. (C) Relationship between rod lengths and amino acid numbers in the rod domain. Data from DDBJ/EMBL-Bank/GenBank (accession, D64087 for DcNMCP1 and accession, AB514509 for DcNMCP2) and chicken lamin B (Heitlinger et al. , 1992) were used. (D) Structural models illustrating NMCP1 and NMCP2 homo-dimers, deduced from electron micrographs of corresponding carrot protein analogues and a structure prediction based on the protein sequence.

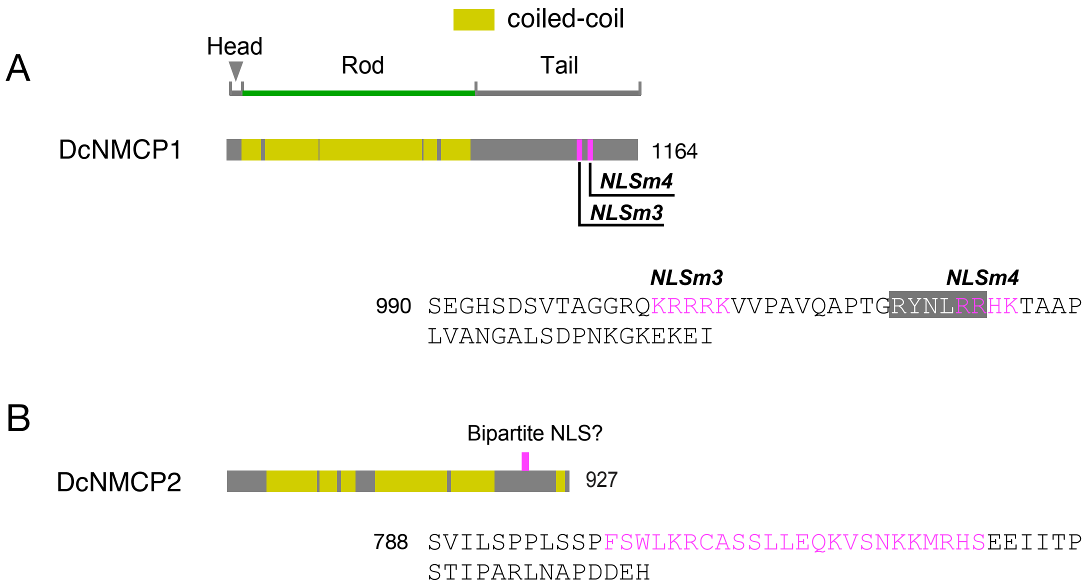

Fig. S4. Carrot NMCP1 (DcNMCP1) and NMCP2 (DcNMCP2). (A) DcNMCP1. (B) DcNMCP2. NMCPs have three domains; an N-terminal head, a central helical rod, and non-helical C-terminal tail domains. The rod domain is composed of segmented coiled-coil regions. The C-terminal domain is predicted to form a globular structure. The C-terminal domain of NMCP1 contains a conserved RYNLRR region (grey background) overlapping with a nuclear localization signal (NLS) (magenta). The C-terminal domain of DcNMCP2 lacks the RYNLRR sequence and monopartite NLS motifs. DcNMCP2 has a potential bipartite NLS motif in its C-terminal domain.
